# Advances in Understanding Neuropathogenesis of Rift Valley Fever Virus

**DOI:** 10.1146/annurev-virology-091919-065806

**Published:** 2022-09-29

**Authors:** Kaleigh A. Connors, Amy L. Hartman

**Affiliations:** Center for Vaccine Research, School of Medicine; and Department of Infectious Diseases and Microbiology, School of Public Health, University of Pittsburgh, Pittsburgh, Pennsylvania, USA;

**Keywords:** Rift Valley fever, viral encephalitis, arbovirus, bunyavirus, aerosol, rat, monkey, African green monkey

## Abstract

Rift Valley fever virus (RVFV) is an emerging arboviral pathogen that causes disease in both livestock and humans. Severe disease manifestations of Rift Valley fever (RVF) in humans include hemorrhagic fever, ocular disease, and encephalitis. This review describes the current understanding of the pathogenesis of RVF encephalitis. While some data from human studies exist, the development of several animal models has accelerated studies of the neuropathogenesis of RVFV. We review current animal models and discuss what they have taught us about RVFV encephalitis. We briefly describe alternative models that have been used to study other neurotropic arboviruses and how these models may help contribute to our understanding RVFV encephalitis. We conclude with some unanswered questions and future directions.

## INTRODUCTION

Rift Valley fever (RVF) is an emerging arboviral disease that affects livestock and humans in Africa, the western Indian Ocean, and the Middle East. RVF disease was first described in 1931 after sudden mortality in lambs occurred on a farm in the Rift Valley region of Kenya (1). The causative agent, eventually identified and named Rift Valley fever virus (RVFV), has contributed to outbreaks in livestock and humans in the years since with increasing frequency due to climate change (2).

**Figure 1:**
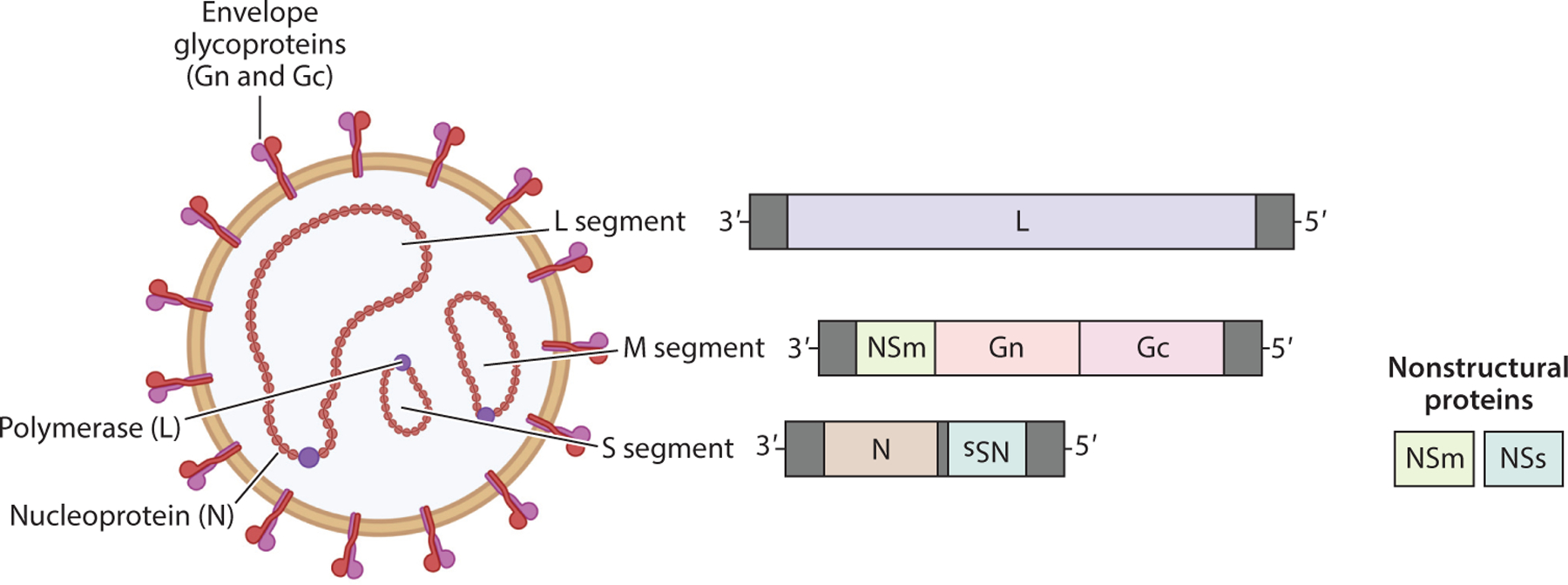
Structure and genome organization of Rift Valley fever virus (RVFV). The envelope glycoproteins Gn and Gc form heterodimers on the virion surface embedded within the lipid bilayer envelope. Within the virion, the three genome segments (L, large; M, medium; and S, small) are complexed with nucleoprotein to form individual ribonucleocapsids. Complementary base pairs on the 3^’^and 5^’^ends of each segment result in circularized nucleocapsids, and the viral polymerase (L) protein associates with nucleocapsids to catalyze transcription upon infection of a cell. RVFV does not contain a matrix protein. Two nonstructural proteins, NSm and NSs, are encoded on the M and S segments, respectively. For RVFV, NSm is encoded 3^’^of Gn and Gc. NSs is encoded in an ambisense orientation on the S segment. Figure adapted from images created with BioRender.com.

RVFV is a member of the *Bunyavirales* order, *Phenuiviridae* family, of negative-sense RNA viruses. RVFV contains three genome segments: The large (L) segment encodes the viral RNA-dependent RNA polymerase (RdRp), the medium (M) segment encodes the surface glycoproteins Gn and Gc along with nonstructural protein NSm, and the small (S) segment encodes the nucleoprotein N and the nonstructural NSs protein (Figure 1) (3). The virus enters cells by receptor-mediated endocytosis, and uncoating of the virion occurs via pH-dependent fusion in the late endosome. The 3 ribonucleocapsids (RNPs) containing the negative-sense genome segments associated with nucleoprotein then undergo transcription into messenger RNA (mRNA) catalyzed by the virus-encoded RdRp. L and S segment mRNAs are translated using free ribosomes, but mRNA from the M segment is translated by endoplasmic reticulum (ER) membrane-bound ribosomes. Replication of (−) genomes to (+) antigenomes and back to (−) genomes is also catalyzed by the RdRp in the cytoplasm of the cell. Newly synthesized RNP segments accumulate at the Golgi where they interact with the cytoplasmic tail of the Gn glycoprotein. Newly assembled virions bud into the lumen of the Golgi, followed by transport to the cell surface in Golgi vesicles and release from the cell.

As an arbovirus, RVFV is spread by several mosquito species that contribute to viral transmission in the enzootic cycle among sylvatic animals and in the epizootic transmission cycle among domestic livestock and people (4). Mosquitoes of the *Aedes* and *Culex* genera are thought to be the primary vectors. Wild animal reservoir(s) such as bats, rodents, or other mammals (gazelles, warthogs, wildebeest, and impala, among others) likely play a role in maintenance and transmission; however, details of RVF circulation in reservoir species remain unknown. Increased rainfall and environmental conditions can promote spread of RVFV into *Culex* mosquitoes and infection of livestock, which display high viremia and severe disease.

RVFV infection of domesticated livestock, which includes sheep, cattle, goats, and camels, commonly results in severe disease where the primary target organ is the liver (5, 6). In ewes, outbreaks of RVFV infection cause large abortion storms, in which up to 100% of fetuses of pregnant animals are lost (7, 8). Human infection occurs by several means: inhalation or exposure of mucous membranes to viral particles following the handling of sick or deceased livestock, consumption of raw animal products, or mosquito bites (9–11).

RVF in humans is often self-limiting; the probability of asymptomatic infection ranges from 90% to 98% (12). Symptoms of acute disease include headaches, body aches, and fever that lasts 3–5 days (13). For some, these acute symptoms can last for a week or more (13, 14). However, 8–10% of symptomatic RVF cases progress to severe disease, which includes hemorrhagic fever, ocular disease, and encephalitis (15–17). No human-to-human transmission of RVFV has been reported.

RVFV is an emerging virus with regular outbreaks and expanding range. Enhanced understanding of the more severe manifestations of RVFV infection is required to develop mitigation and therapeutic strategies. This review describes what is known about one of the more severe outcomes of RVFV infection, neurological disease (meningoencephalitis). We first describe natural disease outcomes in humans followed by the current status of experimental animal models of RVFV encephalitis. Additionally, we briefly describe alternative models of viral encephalitis being employed to study neurological disease and how these models may be applied to the study of RVFV encephalitis.

## EPIDEMIOLOGY

In the past few decades, RVFV appears to be reemerging more frequently, with some regions of eastern Africa experiencing outbreaks of RVF every 4 years (12, 18). A systemic analysis of case reports and seroprevalence studies found that RVFV has been documented in all five African regions encompassing 80% of all African countries (12). The emergence of RVF in the Arabian Peninsula in the early 2000s likely occurred due to the import of infected mosquitos or livestock during trade across the Red Sea (19). Livestock studies show a wide range of RVFV seroprevalence, from less than 1% to nearly 50% in cattle, goats, and camels, and up to 90% of sheep, depending on locality, bioclimatic region, season, breed of animal, and age of animal (20–24). RVFV continues to spread to parts of Africa where it was not previously detected, and introduction of disease into other countries remains a potential threat (12, 25, 26).

Human cases following epizootic outbreaks can range from hundreds to thousands, with case fatalities between 1% and 30% in symptomatic patients reporting to healthcare authorities (15, 17, 27). Human infection most often occurs following outbreaks in livestock (28–30). Incidence of RVFV infection may be underestimated or misdiagnosed unless adjacent to a known livestock outbreak due to the nonspecific symptoms of disease and similarity to cocirculating pathogens (31). RVFV overlaps with other endemic viral diseases with similar disease manifestations, including Lassa fever and West Nile virus (WNV), potentially complicating diagnoses and treatment of cases (32).

## HUMAN CASES OF RIFT VALLEY FEVER PRESENTING WITH NEUROLOGICAL DISEASE

In humans infected with RVFV, a range of clinical disease manifestations can be present in individual patients, many of which involve the central nervous system (CNS). This variety of clinical manifestations makes it difficult to define suspected RVF cases. Incidences of complicated RVFV can be categorized into groups based on symptomology (Table 1). General neurologic manifestations have been observed in up to 17% of cases during some outbreaks (11). In 2000 in Saudi Arabia, 53% of patients who displayed CNS involvement died (11). During acute infection, headaches, neck stiffness, retro-orbital pain, and delirium are among the most common neurological symptoms. However, some patients have a delayed onset of meningoencephalitis that ranges from 5 to 60 days following initial symptoms (13, 15). Delayed-onset complications can include vertigo, disorientation, and hallucinations (13). Vision loss and other ocular manifestations occur at an alarming rate, even in the absence of other serious complications, and are considered a neurological outcome of RVF. During the Saudi Arabian outbreak, 15% of hospitalized patients with severe RVF disease had ocular disease consisting primarily of retinitis and retinal hemorrhage (33). More than 100 outpatients (i.e., patients with otherwise mild illness) reported to healthcare facilities specifically with vision-related complaints including a high rate of anterior uveitis. Chorioretinal scarring was the most frequent long-term effect, and some individuals never regained full vision. Comorbidities such as human immunodeficiency virus and malaria may worsen neurologic symptoms (34). Despite the occurrence of neurologic complications during RVF, there is still little understanding of the mechanisms involved in RVFV pathogenesis in the CNS.

## LABORATORY ANIMAL MODELS OF RIFT VALLEY FEVER VIRUS ENCEPHALITIS

Because neurological manifestations are an outcome of severe RVF in humans, and because autopsy samples from natural human infections are rare, animal models are required to understand neuropathogenesis and clinical outcomes. Several animal models have been developed to specifically study neurologic disease effects in a controlled experimental environment (Table 2). This review focuses on brain involvement and outcomes in animal models of RVFV.

### Rodent Models

Mice are the most common animals used for studies of RVFV pathogenesis due to their extraordinary sensitivity to lethal disease. Unlike most other viruses within the *Bunyavirales* order, wild-type strains of RVFV cause lethal disease in immunocompetent mice of all ages and strains (36, 47–49). The LD_50_ (lethal dose 50) is 1 plaque-forming unit (pfu) administered by most tested routes (subcutaneous, intranasal, aerosol, or intraperitoneal), and the average survival time in most mouse strains is 3–5 days. MBT/Pas mice succumb more quickly [2–4 days post-inoculation (dpi)], while BALB/c mice succumb a few days later (6–8 dpi) (48). Consistent across mouse strains, virus strains, and infection routes is that mice perish due to severe hepatic necrosis, with rampant viral titers in the liver and extraordinary elevations of liver enzymes. The sensitivity to hepatic infection is suspected to be due to the inability of the mouse innate antiviral response to limit viral replication. In BALB/c mice, infection with wild-type RVFV most often leads to lethal disease from hepatitis. However, mice that clear infection from visceral organs succumb to late-onset encephalitis (36, 50). In BALB/c mice inoculated via aerosolization of viral particles, viral encephalitis occurs one day earlier than in mice infected via subcutaneous injection (37). A study of inbred mouse strains (C57BL/6J, 129S1/SvlmJ, NOD/ShiLtJ, A/J, and NZO/HILtJ) demonstrated that infection with even a single virion of wild-type RVFV via footpad injection resulted in severe acute hepatitis as the primary outcome. In three animals that survived acute infection, virus was cleared from the liver but encephalitis developed and led to death (47). Given the inconsistent development of encephalitis in mice, it has been difficult to experimentally study viral encephalitis using these animals. An exception to this general rule has been the use of an attenuated RVFV strain lacking the interfernon (IFN)-antagonist NSs protein (RVFV-DelNSs). When administered intranasally, but not subcutaneously, RVFV-DelNSs-infected C57BL/6J mice develop lethal encephalitis in 7–9 days (35). Additional studies using RVFV-DelNSs have shown that cellular immunity is largely responsible for preventing late-onset encephalitis in subcutaneously infected mice (35, 45, 51, 52). Studies using the Collaborative Cross mouse resource identified several mouse strains that develop lethal neurological disease after footpad inoculation, which would allow more detailed studies of pathogenesis to be conducted using an immunocompetent mouse background (53).

Rats are susceptible to RVFV infection and display a wider spectrum of disease manifestations and outcomes compared with mice (54). Some rat strains succumb quickly to hepatic disease (Wistar-Furth, Brown Norway), while others are much more resistant to disease and death (Lewis, Buffalo, F344) (54, 55). A genetic basis for the disparate disease outcome in rats exists but has not been ascribed to specific genes (56, 57). Aerosol exposure is a reliable inoculation route for inducing encephalitis in most rat strains. August-Copenhagen-Irish and Lewis rats are resistant to disease after subcutaneous inoculation of RVFV, but these animals develop reproducibly lethal encephalitis after aerosol exposure (40). RVFV has been found in the eye of some rat strains infected with RVFV (58), suggesting that ocular disease can be studied using these animals (59).

**Figure 2:**
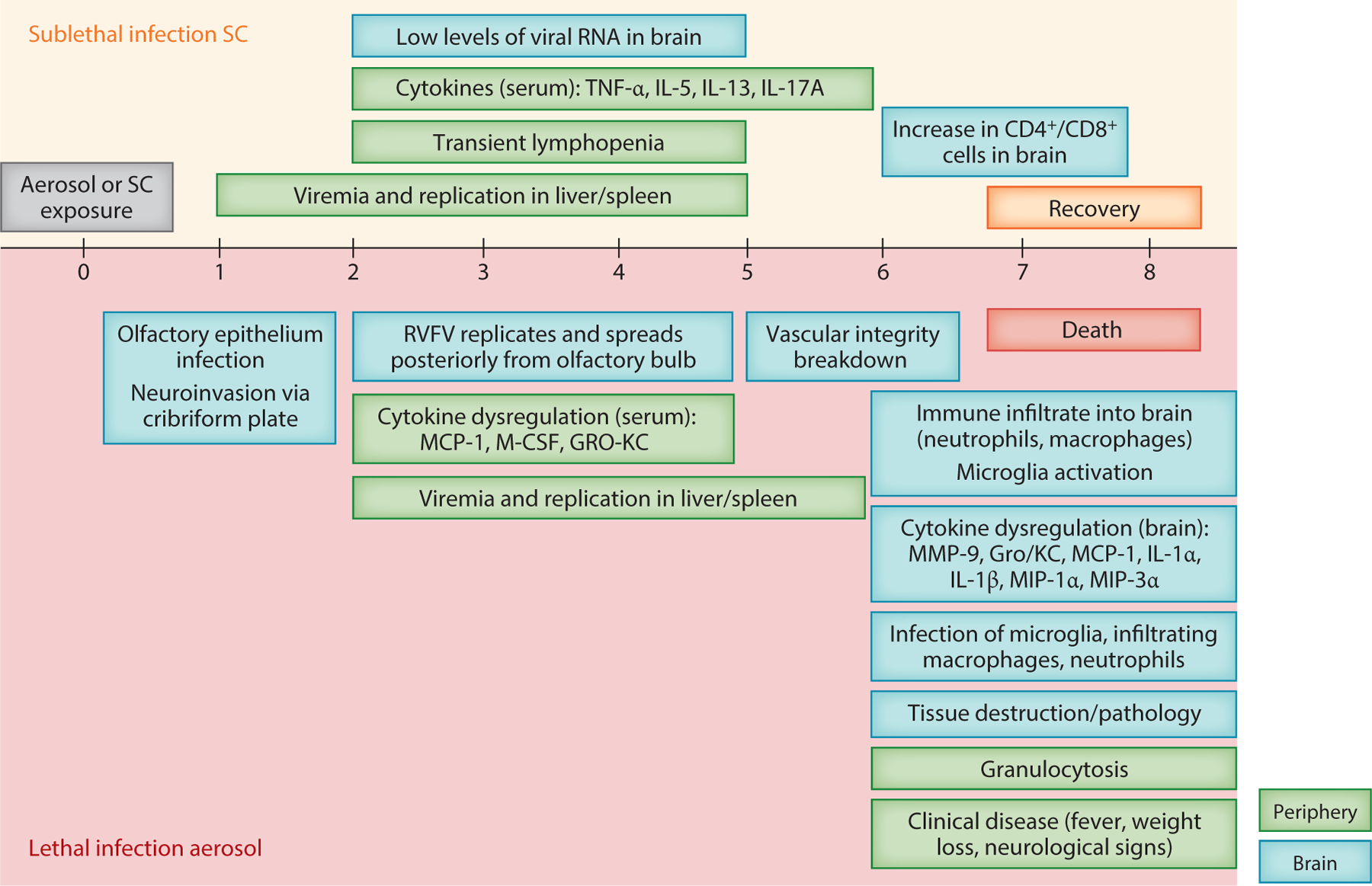
Current model of neuropathogenesis of Rift Valley fever (RVF) in Lewis rats: timing of clinical and virological events after exposure of Lewis rats to Rift Valley fever virus (RVFV). Events above the timeline (*orange background*) are those documented in studies of Lewis rats inoculated by subcutaneous (SC) injection, which represents sublethal infection. Events below the timeline (*red background*) are from animals with lethal neurological disease resulting from aerosol exposure. Events in the periphery are depicted in green, while events in the brain are depicted in blue. (*Top*) After SC infection [lethal dose 50 (LD_50_) > 10^5^ plaque-forming unit (pfu)], rats develop viremia from 1 to 4 days post-inoculation (dpi), during which time the virus replicates in the liver and spleen. Transient lymphopenia occurs along with increases in cytokines in the serum. Low levels of viral RNA can be detected in the brain. However, increases in virus levels in the brain over time are not observed. Modestly increased numbers of T cells are observed in the brain at 6 dpi. Rats display no observable signs of illness. (*Bottom*) After aerosol exposure, the disease course from time of exposure to euthanasia due to severe disease is 7–8 days (LD_50_ = 120 pfu). Cells within the olfactory epithelium become heavily infected, and the virus invades the brain by crossing the cribriform plate into the olfactory bulb. RVFV then replicates to high levels in the neurons of the olfactory bulb and spreads through the cortex and into the cerebellum. Around 5–6 dpi, vascular integrity of the brain is compromised, and immune cells invade from the periphery. These cells consist primarily of macrophages and neutrophils. These cells, along with activated microglia, are infected with RVFV. Inflammatory cytokines are expressed in the brain at high levels during end-stage disease. Death of neurons and disruption of cell architecture of the brain leads to tissue destruction and severe pathology. During this time, the animals develop observable signs of illness, including weight loss, fever, and neurological manifestations (head tilt, circling in cage).

Given the stark contrast in clinical outcomes displayed following subcutaneous and aerosol inoculation of Lewis rats (survival and lethal encephalitis, respectively), Lewis rats have been used for more detailed studies of the pathologic events during RVF encephalitis (Figure 2). Lewis rats succumb to encephalitis 7–8 days after aerosol inoculation with RVFV (ZH501 strain; LD_50_ ~ 120 pfu) (40). After aerosol exposure, cells of the olfactory epithelium are extensively infected, followed by infection of cells across the cribriform plate and subsequent invasion of the olfactory bulb by 2 dpi (60). Microglia become activated during the initial infection of the brain, as the virus replicates to high levels. RVFV spreads from the olfactory bulb through the brain (61). Due to high levels of virus replication, integrity of the brain structure, in particular the glomerular layer of the olfactory bulb and the vascular integrity of the cortex, is compromised after 5 dpi (60, 61). At the time of brain vasculature deterioration (~5 dpi), immune cells (macrophages and neutrophils) flood the vasculature and parenchyma. Activated microglia, infiltrating macrophages, and neutrophils are infected with RVFV based on flow cytometry and immunofluorescence analysis (41). Histological hallmarks of RVFV encephalitis include vasculitis, meningitis, and neuronal apoptosis (58). The chemokines MMP-9, MCP-1, Gro/KC, MIP-1α, MIP-3α, IL-1α, and IL-1β are elevated first in serum at 3–5 dpi and then are found in high levels in the brain after 5 dpi (41, 58). In contrast, Lewis rats infected subcutaneously develop transient viremia and display moderate levels of viral replication in the liver and spleen (2–5 dpi) and mildly elevated cytokines, such as tumor necrosis factor (TNF)-α, in the serum. While viral RNA can be detected at low levels in the brain of subcutaneous-inoculated rats, viral RNA levels do not increase dramatically, as observed in aerosol-inoculated animals. Taken together, studies of Lewis rats infected with RVFV indicate that high levels of virus replication in the brain after aerosol inoculation cause structural and vascular disintegration, promoting rampant inflammation at end-stage disease. Therapeutic interventions for RVF encephalitis will need to consider how to limit viral entry into the brain, and once there, will need to limit both virus replication and inflammation to protect from lethal disease.

### Nonhuman Primate Models

While nonhuman primates (NHPs) can be infected with RVFV, they are far less susceptible to disease than are rodents and, as such, are more similar to humans in that most disease is mild. In rhesus monkeys inoculated intravenously, intramuscularly, or subcutaneously with RVFV, ~20%develop signs of hemorrhagic fever and ~40% show mild signs of disease such as fever, rash, or vomiting, while the remaining show no clinical disease (62–64). In most infected animals, transient viremia coincides with fever. Rhesus and cynomolgus macaques exposed to aerosols containing RVFV develop transient viremia, fever, and decreased appetite (42, 44, 65). Lack of reproducible severe disease in these species makes it difficult to study viral pathogenesis and efficacy of countermeasures using these animals.

Reproducible lethal disease has been documented in common marmosets exposed to RVFV via intravenous, subcutaneous, and intranasal routes, which lead to both hemorrhagic fever and delayed-onset encephalitis (44). Marmosets inoculated intranasally display 100% mortality with neurological signs 8–11 dpi (44). Both marmosets and old-world African green monkeys (AGMs) are susceptible to neurological disease after aerosol RVFV infection, while cynomolgus and rhesus macaques develop mild febrile illness (42). Beginning 8–10 dpi, both marmosets and AGMs develop symptoms of neurologic disease, including drooling, ataxia, head pressing, and seizures, and the animals succumb 10–12 dpi (42). At end-stage disease, AGMs contain infectious virus only in CNS tissues, while marmosets contain virus in the CNS and abdominal viscera. High numbers of granulocytes predominate in complete blood count analyses at end-stage disease in both monkey models. Infectious virus and viral RNA are found within the eyes, although no pathologic changes in the eye have been documented. Fever is a dominant characteristic in both species, with pyrexia being biphasic in marmosets and monophasic in AGMs. Widespread lesions in the brain with moderate numbers of inflammatory cells (lymphocytes and neutrophils) occur in marmosets and AGMs. Neuronal necrosis and neuronophagia (phagocytosis of degenerate and necrotic neurons) are also commonly observed. There is intense multifocal positive staining of neurons for RVFV antigen by immunohistochemistry. The reproducible encephalitic disease in both marmosets and AGMs makes them useful for studies of neuropathogenic mechanisms.

**Figure 3:**
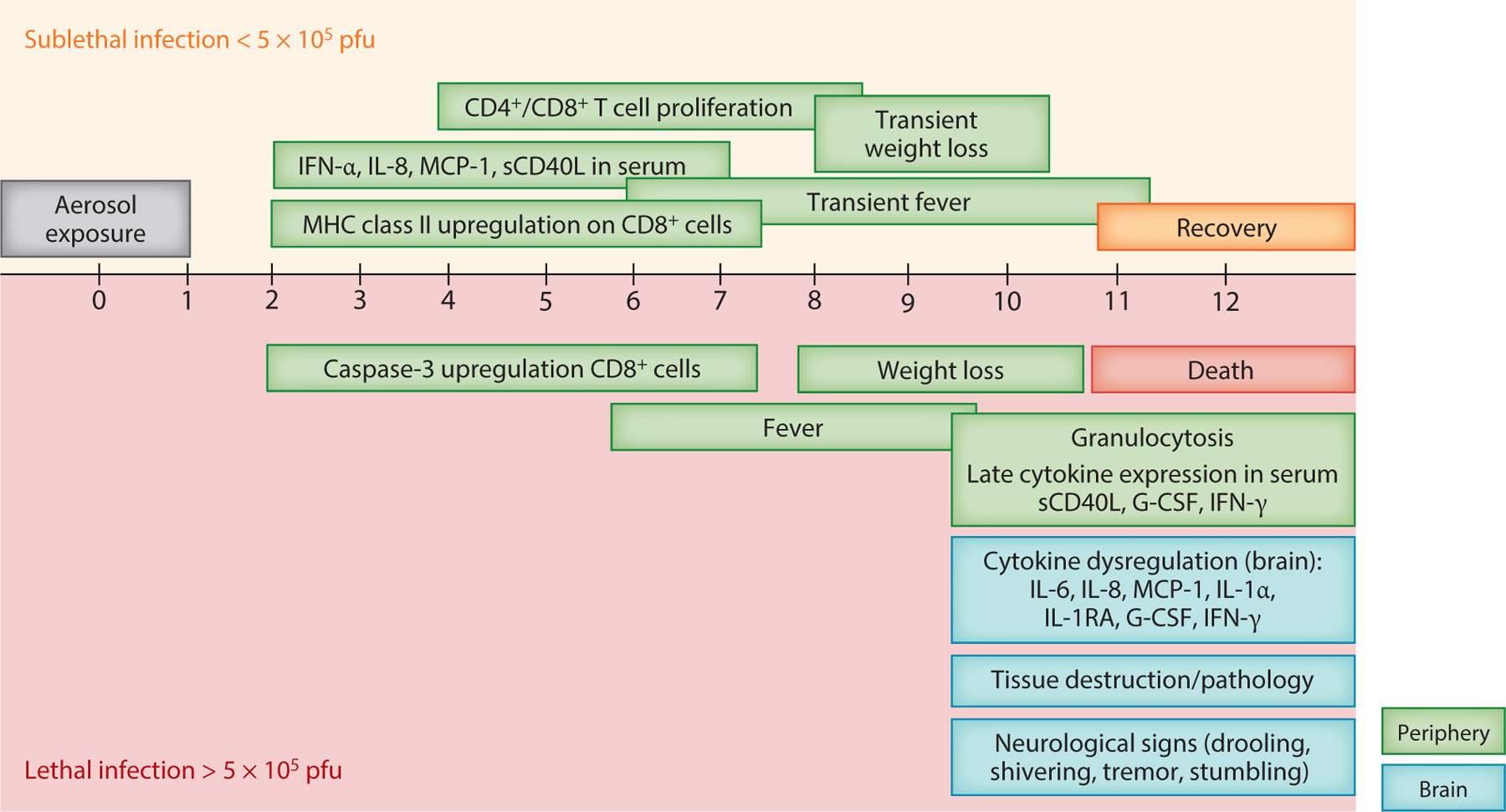
Current model of neuropathogenesis of Rift Valley fever (RVF) in African green monkeys (AGMs): timing of clinical and virological events after aerosol exposure to RVFV. The lethal dose 50 (LD_50_) of RVFV in AGMs is 5 ×10^5^ plaque-forming unit (pfu). Events above the timeline (*orange background*) are those documented in RVFV-infected AGMs that survive aerosol infection; events below the timeline (*red background*) are from animals with lethal neurological disease. Events in the periphery are depicted in green, while events in the brain are depicted in blue. The disease course from time of exposure to euthanasia due to severe disease in AGMs is 9–12 days. (*Top*) In animals that survive infection, an early cytokine response is detectable in the serum. There is an increase in major histocompatibility complex (MHC) class II expression by CD8^+^ T cells as early as 2 days post-inoculation (dpi), followed by an increase in both CD4^+^ and CD8^+^ T cell numbers beginning at 4 dpi. Surviving AGMs may develop transient fever and weight loss, but these animals recover by 10–12 dpi. (*Bottom*) In lethally infected animals, there is an absence of an early cytokine response and immune activation in the peripheral blood. Instead, CD8^+^ T cells express activated forms of caspase-3. AGMs develop severe fever beginning at ~ 6 dpi, followed by weight loss and neurological manifestations (excess salivation, tremors, and stumbling). High numbers of granulocytes in the blood are detectable at ~10 dpi and are a hallmark of severe disease. AGMs reach euthanasia criteria between 9 and 12 dpi and have high levels of inflammatory cytokines, viral titers, and severe pathology in the brain.

A more in-depth immunological analysis of AGM compared lethal and sublethal infections (43). AGMs that survive aerosol exposure to RVFV mount an early IFN and cytokine response, along with increased numbers of CD4^+^ and CD8^+^ T cells and activation markers. In contrast, AGMs that succumb to neurologic disease have an absent IFN response and no increase in T cell numbers or activation markers; instead they have increased caspase-3 expression in CD8^+^ T cells. Similar to the situation in RVFV-infected rats, the brains of encephalitic AGMs contain high levels of MCP-1, MIP, IL-6, and IL-8 at end-stage disease. The current model of RVFV neuropathogenesis in AGMs is shown in Figure 3. Collectively, the AGM model highlights the importance of an early innate response and induction of cellular immunity as key predictors of survival (43, 52).

### Other Animal Models

Gerbils exposed to RVFV subcutaneously display dose- and age-dependent development of encephalitis, with up to 100% mortality in young gerbils with little liver disease (46). However, when a low dose (50 pfu) of RVFV is administered intracranially, all animals, regardless of age, succumb to lethal encephalitis at 7 dpi. Ferrets are susceptible to RVF encephalitis. Exposure of ferrets to RVFV by high-dose intranasal inoculation results in lethal febrile disease with clinical and pathological manifestations of encephalitis by 8–11 dpi (66). Inflammation occurs in the brain and the choroid of the eye. Elevations of liver enzymes (ALT, AST) are observed in lethally infected ferrets. Thus, ferrets may serve as an attractive large animal alternative to NHPs for studies of RVFV pathogenesis, which are more expensive and difficult to acquire.

## COMMON PATHOLOGICAL OBSERVATIONS

Human brain tissue from autopsied RVFV cases is not often available, but histological samples were obtained during outbreaks in South Africa (1974), Saudi Arabia (2000), and Kenya (2006) (6, 67). Histology of human brain lesions reveals focal necrosis alongside infiltration of lymphocytes and macrophages with perivascular cuffing (67). Encephalitis also has been documented in RVF cases in individuals with hemorrhagic fever, suggesting brain involvement in this severe form of RVF (68). Common in rat, ferret, and NHP models of RVFV neurologic disease are high titers of virus within the brain itself, likely contributing directly to death of neurons, inflammatory responses (such as MCP-1, Gro/KC/IL-8, IL-6, IL-1α, IL-1RA, G-CSF), and infiltration of macrophages and neutrophils, which are seen in all animal models. Primary histological features in animal models of neurologic disease are perivascular cuffing (inflammatory cells around blood vessels within the brain), meningitis (leukocytic cells within the meninges), encephalitis (lymphocytes, macrophages, neutrophils within the brain parenchyma), and neuronal necrosis or apoptosis. All animal models that manifest neurologic disease also contain virus within the eye, an aspect of RVF pathogenesis that has remained unexplored.

## CELL CULTURE AND ALTERNATIVE MODELS OF VIRAL ENCEPHALITIS

Several brain-derived cell lines are highly susceptible to RVFV infection in vitro. Viral replication of the attenuated MP-12 RVFV stain is most efficient in brain cell lines derived from North American livestock and wildlife compared with cell lines derived from kidney and lung (69). The human neuroblastoma cell line SH-SY5Y and microglia cell line HMC3 as well as rat microglia cell line HAPI support high levels of viral replication following infection with the wild-type ZH501 RVFV strain (41). In addition to immortalized cell lines, RVFV replicates in primary cultures of rat neurons and glial cells (70, 71). These studies highlight the in vitro susceptibility of CNS cells to RVFV infection.

While animal models are exceedingly useful in studies of viral pathogenesis, 2D and 3D cell models can be informative. These include brain organoids and ex vivo tissue-culture models. Brain organoids are 3D in vitro culture systems that mimic the organizational and developmental processes of the brain (72). Induced-pluripotent stem cell (iPSC) organoids contain a spectrum of developing neurons, exhibiting an inside-out patterning that includes immature neurons that migrate radially away from the neurogenic region to integrate into the tissue as mature neurons (73). Organoids were used to show that Zika virus (ZIKV) infection leads to loss of the ventricular zone and neuronal layers, mimicking microcephaly observed in ZIKV infection of developing fetuses (74, 75). Brain organoids also have been used to study La Crosse virus (LACV), a neurotropic orthobunyavirus related to RVFV (76, 77). LACV infection of brain organoids decreases structural complexity by inducing loss of cells undergoing neuron development (77).

An alternative 3D model is the organotypic brain-slice culture model, which is used to study molecular and cellular brain functions and neurodegenerative disorders, including Alzheimer disease. Brain-slice cultures allow cell-cell interactions and structural integrity to be somewhat maintained, while preventing influence of peripheral infiltrating cells, such as leukocytes (78). Brains are typically obtained from young postnatal mice or rats, sectioned into 100–500 μm thickness, and maintained in culture for extended periods (79, 80). These organotypic-slice cultures have been used to study other neurotropic arboviruses, including WNV and ZIKV (81–83), offering insight into neuropathogenesis of these viruses in a physiologically relevant ex vivo system. Neither organoid nor brain-slice cultures have yet to be reported for the study of RVFV, but these models may allow important experimental questions to be answered.

## CONCLUSIONS AND UNANSWERED QUESTIONS

Many unanswered questions remain despite significant progress in understanding mechanisms underlying RVFV neuropathogenesis. While there is now a better grasp of how RVFV causes lethal disease after aerosol exposure in Lewis rats, we do not know how the virus gets to the brain after peripheral infection and why Lewis rats survive subcutaneous infection despite this fact. Data from studies of Lewis rats and AGMs highlight the importance of induction of early innate and adaptive immune responses in controlling disease. In addition, the mechanism by which RVFV infection results in delayed-onset encephalitis has not been explored using any animal model. The development of additional animal models that reliably allow RVFV neuroinvasion following peripheral inoculation would assist in answering some of these questions. The ocular disease aspect of RVFV pathogenesis is poorly understood and deserves consideration.

Importantly, the neurological animal models described in this review have not been used to investigate the efficacy of therapeutic interventions. Now that rat and monkey models of RVF encephalitis have been characterized to some extent, vaccines and therapeutics should be tested for efficacy in these animals. Recognizing that prevention or amelioration of virus replication in the brain is difficult, approaches such as mucosal (intranasal) vaccination could be tested for the capacity to prevent disease following inoculation by peripheral, intranasal, or aerosol routes. Targeting of therapeutic drugs to the CNS, while difficult, also could be evaluated using these models.

Finally, limited data exist on the basic biology of RVFV infection in the CNS. The role of resident CNS cells—including neurons, microglia, and astrocytes—during RVFV infection and pathogenesis remains understudied. Using alternative models such as cerebral organoids or organotypic brain-slice cultures, in addition to animal models, could shed light on the contributions of resident brain cells during RVFV disease.

## Figures and Tables

**Table 1:** Potential clinical classifications of Rift Valley fever disease and their corresponding neurological features

Disease outcome	Primary clinical feature(s)	Neurological features
Acute disease (uncomplicated)	Fever, chills, nausea, vomiting, diarrhea, abdominal pain, body aches, headache	Not applicable
Severe hemorrhagic fever disease with concomitant neurological involvement	Jaundice, rash, vomiting blood, blood in urine/stool, nosebleed, conjunctival hemorrhage	Headache, neck stiffness, retro-orbital pain, hypersalivation, teeth grinding, confusion, stupor, coma
Acute disease with concomitant neurological signs	Acute disease	Neck stiffness, retro-orbital pain, hypersalivation, teeth grinding, confusion
Acute disease followed by delayed-onset neurological disease	Acute disease	Recurring headache, confusion, neck stiffness, disorientation, vertigo, hallucination
Ocular disease	Acute disease	Vision loss, scotomas, retinal lesions, retinitis, retinal hemorrhage, uveitis

**Table 2:** Laboratory animal models of Rift Valley fever neurologic disease

Species/Strain	Rift Valley fever virus strain; route of infection	Neurological manifestation(s)	Reference(s)
Mice			
C57BL/6J	RVFV-DelNSs; IN	Early-onset neurologic signs, development of meningoencephalitis	35
BALB/c	ZH501; SC	Delayed-onset encephalitis in mice surviving acute liver disease	36
	ZH501; AERO	Earlier neuroinvasion in AERO-infected mice compared to SC infection	37
	ZH501; IC	Rapid development of neurologic symptoms including paralysis and convulsions	38
STAT-1 knockout	MP-12; IN	Cage circling, head pressing	39
Rats			
Lewis (LEW/SsNhsd)	ZH501; AERO	Circling or rolling in cage, head tilt, unsteady gait	40,41
August-Copenhagen-Irish (ACI/SegHsd)	ZH501; AERO	Circling or rolling in cage, head tilt, unsteady gait	40
Nonhuman primates			
African green monkey *(Chlorocebus aethiops)*	ZH501; AERO	Fever, drooling, ataxia, horizontal nystagmus, head pressing, seizures	42,43
Marmoset *(Callithrix jacchus)*	ZH501; AERO	Biphasic fever, ataxia, tremors, seizures	42,44
	ZH501; SC, IN	Late-onset encephalitis in SC inoculation; lethal encephalitis in all IN-inoculated animals	44
Other			
Ferret *(Mustela purotius furo)*	ZH501; ID, IN	Fever, head tilt (ID), ataxia, seizures/tremors, hind-limb weakness (IN)	45
Gerbil *(Meriones unguiculatus*)	ZH501; SC, IC	Hind-limb paralysis; focal necrotizing encephalitis observed by IHC	46

Abbreviations: AERO, aerosol; IC, intracranial; ID, intradermal; IHC, immunohistochemistry; IN, intranasal; SC, subcutaneous.
